# Enzyme-Triggered Crosslinked Hybrid Hydrogels for Bone Tissue Engineering

**DOI:** 10.3390/ma15186383

**Published:** 2022-09-14

**Authors:** Ankur Sood, Seong Min Ji, Anuj Kumar, Sung Soo Han

**Affiliations:** 1 School of Chemical Engineering, Yeungnam University, 280 Daehak-ro, Gyeongsan 38541, Korea; 2 Institute of Cell Culture, Yeungnam University, 280 Daehak-ro, Gyeongsan 38541, Korea

**Keywords:** biomaterials, bone tissue engineering, crosslinking, enzyme, hydrogels, polymers

## Abstract

The quest to develop state-of-the-art hydrogels for bone tissue engineering has accompanied substantial innovation and significant progression in the field of bioactive hydrogels. Still, there is scope for advancement in this cell-friendly and biocompatible scaffold system. The crosslinking approaches used for hydrogel synthesis plays a decisive role in guiding and regulating the mechanical stability, network framework, macroscopic architect, immunological behaviors, and cellular responses. Until recently, enzyme-based crosslinking strategies were considered as the pinnacle in designing efficient hybrid hydrogel systems. A variety of enzymes have been explored for manufacturing hydrogels while taking the advantage of the biocompatible nature, specificity, ability to produce nontoxic by products and high efficiency of enzymes. The current review focuses on the utility of different enzymes as crosslinking agents for hydrogel formation with their application in bone tissue engineering. The field of enzyme crosslinked hydrogel synthesis is rapidly maturing with a lot of opportunities to be explored in bone tissue engineering. Enzyme-based in situ and externally crosslinked hydrogels for bone regeneration is an attractive field, and with innovation in using engineered enzymes this field will continue to flourish with clinical orientation.

## 1. Introduction

The self-remodeling and regeneration of bone tissues is a unique feature, but this ability is restricted to certain situations of orthopedic ailments where the injured part of the bone is incapable of healing on its own [1,2]. A complex architect of bone tissues establishes the diversity of impairments caused to these structures [3]. The reparation of bone tissues embraces a sequence of events and involves many cells in a well-choreographed and regulated fashion [4]. Over the past few decades, enormous progress has been made in bone reconstruction techniques, which include scaffold-based tissue engineering [5], the delivery of growth factors through advanced delivery systems [6], and the fabrication of bioactive materials (biomaterials, polymers, patches, hydrogels etc.) [7,8,9] to stimulate the growth of tissues/cells and adjacent microenvironment.

For a proficient bone regeneration approach, the designed scaffold should be nontoxic, biocompatible, support cell attachment and proliferation, and, most importantly, it should support the progression of an extracellular matrix (ECM) in the injured tissue [10]. The ECM of a bone plays a vital role in the reparation of the injured tissues and possesses a hierarchical architect and its decisive aspiration is to imitate the native tissue anatomy, chemical constitution, and physiological attributes while designing a scaffold for bone regeneration [11,12]. Constant efforts are being made to design more biomimetic scaffold systems with the aim to reconstruct the bone matrix in its native form. Of several approaches, hydrogels have gained the considerable interest of many researchers as a persuasive platform for bone tissue engineering applications [13,14].

A hydrogel is three-dimensional network composed of crosslinked natural or synthetic macromolecules with an ability to absorb an enormous quantity of water or biological fluids with a close resemblance to biological tissues [15]. Hydrogels are capable of cell attachment, which is influenced by their ability to efficiently exchange oxygen and bioactive molecules across their networks, thus providing structural and physiological support for cell growth and proliferation [16]. Moreover, the pharmaceutically active molecules which could be transported by hydrogels helped in cell functioning and regulation in situ. In order to prepare hydrogels, various approaches are explored through major advances, including chemical and physical strategies for hydrogel formation [17,18]. While physical hydrogels are mainly concentrated upon the exposure of external stimuli, crystallization, and ionic/hydrophobic interactions for gelation [19,20], the chemical approach requires the use of chemical crosslinkers or reactions to trigger gel formation. The physical approaches for hydrogel formation may results in weak mechanical and physiological stability in vivo but the non-usage of harmful chemical agents is an advantage to this approach. On the other hand, mechanical and physiological superiority, along with strong bonding, are advantages for chemical crosslinking strategies. Until recently, enzyme-based crosslinking strategies had been suggested as promising crosslinking approaches with minimal crosslinker requirements, high specificity, and non-toxic byproducts [21,22]. Enzymatic reactions are mostly catalyzed at a neutral pH and in an aqueous environment in moderate conditions, giving them an edge over other approaches [23]. The field of enzyme-based crosslinking has been keenly explored for various biomedical applications. However, enzyme-crosslinked hydrogels targeting bone tissue engineering is a fairly new field with many opportunities yet to be explored. There a number of reviews focusing on enzyme-crosslinked hydrogels and their biomedical applications [22,24] with an emphasis on tissue engineering in general [21,25]. There have been reviews on physical and chemical crosslinked hydrogels for bone tissue engineering [4,26]. However, to date, a dedicated review of bone tissue engineering specifically regarding the aspect of enzyme-crosslinked hydrogels is missing.

The current review focuses on the utility of five different enzymes that are being explored as crosslinkers for hydrogel formation in bone tissue engineering. In this review a brief outline of different crosslinking strategies is discussed with their advantages and disadvantages in hybrid hydrogel formation. Further, a framework of the mechanistic basis of enzyme-triggered crosslinking in hydrogel formation is discussed. In the following section, different enzymes are highlighted with their utility in regard to hydrogel formation in bone tissue regeneration. Finally, the review is concluded with the current challenges that need to be overcome while using enzyme-based crosslinking approaches with a spotlight on their future implications.

## 2. Hybrid Hydrogels

Hydrogels are 3D hydrophilic polymeric networks that can absorb a high level of water molecules and swell while upholding their structure due to the physical or chemical crosslinking of distinctive polymeric chains. Hydrogels exhibit stimuli-responsive behavior in a particular environment due to their functional groups with specific charges [27]. Hybrid hydrogels are denoted to as hydrogel-networks, which are morphologically, chemically, and functionally distinct building blocks interlocked physically or chemically. Depending on the nature and the size of the building blocks (components), the hybridization might occur at a microscopic scale or on a molecular level. The modular type of the design, united with the likely synergistic effect of the hybrid coordination, leads to novel hydrogels with multifunctional characteristics [28]. Therefore, hybrid hydrogels are characteristically heterogeneous and are effective for cell–matrix interactions in tissue engineering applications [29,30]. Due to their adaptable characteristics, hybrid hydrogels have been increasingly receiving scientific attention for different biomedical applications, particularly bone tissue regeneration.

The characteristics of hybrid hydrogels are greatly associated with their crosslinking reaction mechanisms, and thereby the functional development of hydrogels is particularly dependent on novel manufacturing approaches. In addition, a balanced combination of physical and chemical crosslinking methods is promising in creating hierarchical and/or stimuli-responsive hydrogels for bone tissue regeneration [31]. Therefore, for an effective design strategy, an understanding of the crosslinking reaction mechanisms is highly desired. In the next section, we discuss the various crosslinking approaches for preparing hybrid hydrogels as well as their advantages and disadvantages.

## 3. Various Crosslinking Approaches for Developing Hybrid Hydrogels

**Figure 1:**
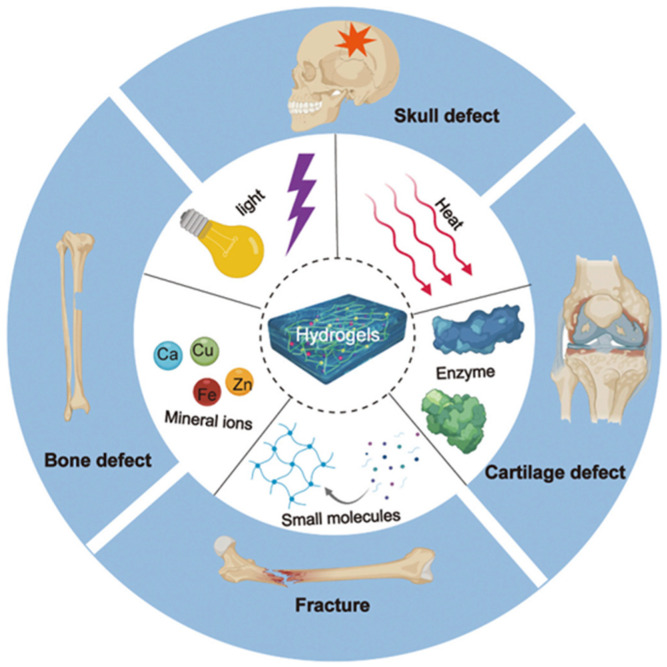
Diverse bionic hydrogels prepared by various activated environments (e.g., temperature (heat), light, mineral ions, small bioactive molecules, enzymes, etc.) for bone tissue regeneration. Reproduced from [4].

Recently, various crosslinked environments, including physical and chemical, have been utilized to develop hybrid hydrogels to accelerate bone tissue restoration ability [18,31]. Physically crosslinked hydrogels involve the stimulation of chain crosslinking using light or temperature, whereas chemically crosslinked hydrogels include the covalent or coordinate interaction between the polymeric chains to construct durable hydrogel-network structure through molecule or ionic crosslinking agents [32]. In addition, physically crosslinked hydrogels are promising due to their mild gel-forming parameters (e.g., temperature, low toxic crosslinking effect) and could be used in bone defects with small cavities, while chemically crosslinked hydrogels could be employed in hard and sizeable bone defects. The formation of hydrogels for biomedical applications should be able to address some key requirements in terms of biomedical applications that include substantial stability when exposed to different microenvironments (based on targeted application), minimal cytotoxicity, and negligible immunological suppression. In fact, efforts are being made to develop hybrid hydrogels that act as immunomodulators. The careful consideration of these aspects is a foremost requirement for achieving the clinical translation of the fabricated hybrid hydrogels. Diverse bionic hydrogels have widely been prepared by using various crosslinked conditions for bone tissue regeneration, as can be seen in Figure 1 [4].

### 3.1. Physically Crosslinked Hydrogels

Physically crosslinked hydrogels are typically prepared by inter-molecular reversible interactions, such as hydrogen bonds, electrostatic (ionic) interactions, and hydrophobic/hydrophilic interactions, the entanglements of polymeric chains, stereocomplex crystallization, metal coordination, and the stacking of π–π bonds. These hydrogels are more efficient in biomedical areas, which prevent potential cytotoxicity as they use no chemical crosslinking agents. Additionally, they are stimuli-responsive to the environment and exhibit injectability and self-healing abilities at room temperature [4,27,31]. Some selective crosslinking approaches are described.

### 3.2. Chemically Crosslinked Hydrogels

**Figure 2:**
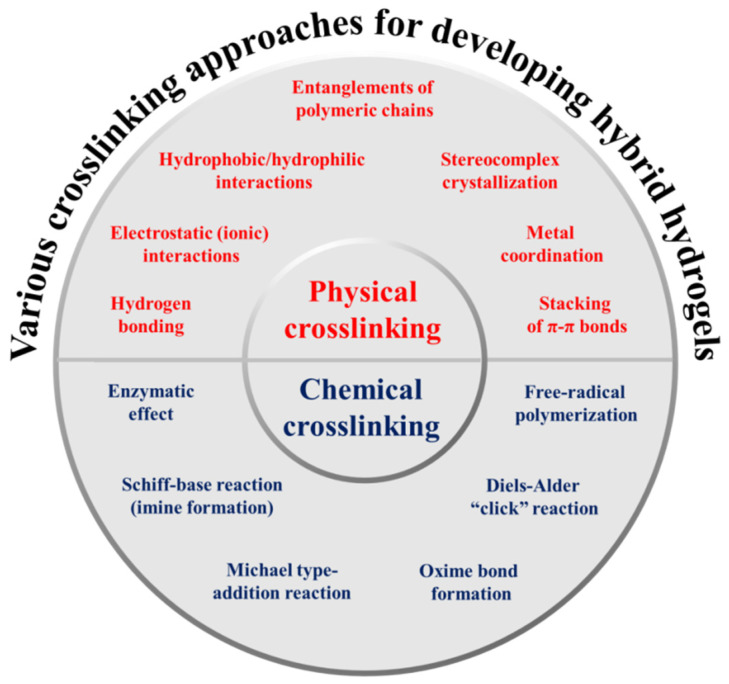
Various potential crosslinking approaches utilized for manufacturing hybrid hydrogels.

Chemically crosslinked hydrogels are usually prepared by forming covalent bonds among polymeric chains; they are naturally strong and permanent linkages as compared to physically crosslinked hydrogels. This includes, the enzymatic effect, free-radical polymerization, Schiff-base reaction, Michael type-addition reaction, oxime creation, and Diels–Alder “click” reaction. Chemically crosslinked hydrogels are more stable (under physiological environment) by exhibiting remarkable mechanical performances and tunable degradation profile [4,27,31]. Various potential crosslinking approaches have broadly been utilized for manufacturing hybrid hydrogels, as shown in Figure 2 and their brief summary with advantages and disadvantages is provided in Table 1.

## 4. Enzyme Crosslinking Approaches for Bone Tissue Engineering

Crosslinking strategies have been extensively explored in order to design novel hydrogels for bone tissue engineering. Lately, the fabrication of hydrogels with the help of enzyme-mediated crosslinking approaches has been extensively explored. This approach has resulted in promising outcomes with convincing prospects [61]. Enzymes are required in minimal quantity and are very efficient in their actions, as they increase the reaction rate without being expended during the course of the reaction process. The efficiency of an enzyme is defined by the number of substrate molecules converted into products per unit of enzyme, which is also known as turnover number (k cat). The high efficiency of enzyme-based reactions comes from the precise specificity, which ensures the conversion of a particular type of substrate to products [62]. So far, many enzymes have been explored in order to prepare biomimetic hydrogels for bone tissue engineering. The details of every enzyme-based crosslinking approach are discussed in the following sections.

### 4.1. Tyrosinase

**Figure 3:**
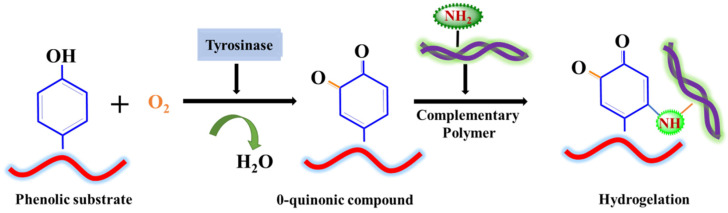
Mechanism of action of tyrosinase.

Of all the other enzymes, tyrosinase has been most often explored for various tissue engineering applications, including bone tissue engineering. In the case of mammals, the tyrosinase is located in melanosome, which manufactures melanin [63]. The mechanism of the action of tyrosinase is based on the oxidation of the phenolic groups (tyrosine, catechol, and polyphenols) present in their active sites, without the prerequisite of any cofactors. Due to the abundance of these phenolic groups in human proteins, they can be easily conjugated to hydrogels, making them efficient players in crosslinking strategies [22,64] (Figure 3). Moreover, the mechanism of the oxidation reaction of tyrosinase strongly resembles the cascade of events in the 3,4-dihydroxy-l-alanine (DOPA) induced mussel adhesion on sea rocks in mussel foot protein (Mfp), which has been extensively explored in tissue engineering and regenerative medicines [65].

**Figure 4:**
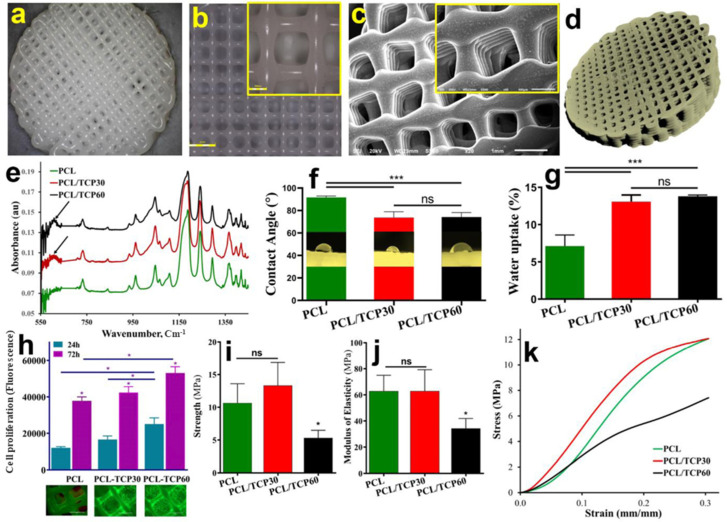
Characterization of 3D-printed functionally graded scaffolds. (**a**) Optical image of the 3D-printed scaffold; (**b**) 3D laser microscope images (scale bars: 2 mm and 500 μm); (**c**) SEM images (scale bars: 1 mm and 500 μm); (**d**) μCT image; (**e**) FTIR spectra of polycaprolactone (PCL) and composite scaffolds; (**f**) water contact angle values of different scaffolds; (**g**) relative water absorption of various scaffolds; (**h**) cell viability and proliferation on scaffolds at different time points with Live/Dead staining data at the bottom (Scale bar: 1000 μm); (**i**) compressive strength, (**j**) elastic moduli, and (**k**) stress–strain curve of scaffolds. PCL: beta-tricalcium phosphate (TCP): 1,6-hexanediamine (HAD), *: *p* < 0.05, ns: non-significance, ***: *p* < 0.001, s: significant. Reproduced with permission from [72]. Copyright 2021 Elsevier.

Inspired by mussels, Sousa et al. fabricated a dopamine moiety on the surface of hyaluronic acid to develop freestanding multilayer membranes using layer by layer technology to enhance the cell adhesion and interaction between the construct and cells [66]. The mechanical attributes and enhanced adhesion were examined and found to be optimal for bone tissue engineering. Further, Mishra et al. demonstrated the manufacturing of injectable ink, comprising of carboxymethyl-chitosan (CMC)/gelatin/nano-hydroxyapatite (nHAp) via tyrosinase/p-cresol-mediated in situ crosslinking [67]. The study highlighted the degree of crosslinking as a prime factor to uncover the differentiation and proliferation of osteoblast cells along with the stability of in situ formed gels in vivo. In another study, Sharma et al. fabricated tyrosinase-crosslinked silk fibroin and gelatin-mixed hydrogel doped with calcium ions. The study aimed to investigate the rheological and calcium releasing behavior of the fabricated hydrogel in bone tissue regeneration. The work also highlighted the ability of the designed hydrogels in augmenting the osteogenic differentiation of human bone marrow-derived mesenchymal stromal cells [68]. A similar strategy was explored by Chameettachal et al. to fabricate tyrosinase-crosslinked silk fibroin and gelatin hydrogels to explore the chondrogenic differentiation and suppression of hypertrophic differentiation across different cell modalities for chondrocytes and mesenchymal progenitor cells (hMSCs) [69]. The combination of silk fibroin and gelatin has been reported by other research group for bone tissue engineering [70]. In another study, tyrosinase-mediated chitosan/gelatin and chitosan/gelatin/nanohydroxyapatite hydrogels were synthesized for bone tissue engineering [71]. Rapid and permanent gelation was the hallmark of this work. Here, derivatized tyrosinase (mTyr-CNK)) was engineered to exhibit a high catalytic efficiency for tyrosine/DOPA-tethered hydrogels across a broad pH range. The obtained hydrogels exhibited good porosity with high swelling ratios. Further, in order to enhance the interfacial adhesion of an osteo-mucosal construct, a study reported tyrosinase-based adhesion approach was presented that resulted in healthy tissue regeneration along with the enhanced adhesion for the soft/hard components of designed constructs [72]. The data obtained from the study confirmed the enhanced proliferation of osteoblast cells via aminolysis and improved osteoblast cells differentiation via tyrosinase present in collagen. Furthermore, the evidence of multilayered epithelium on the osteo-mucosal model with viable fibroblasts and osteoblasts was also demonstrated (Figure 4). The fabricated construct could find clinical implications as a graft material in surgeries and can serve as an in vitro model to investigate the applicability of dental materials. All these studies reflect the elevated adhesion capacity of tyrosinase. The gentle and fast crosslinking process of tyrosinase-based crosslinking in an aqueous environment further aids in its widespread utility in bone tissue engineering.

### 4.2. Lysozyme

**Figure 5:**
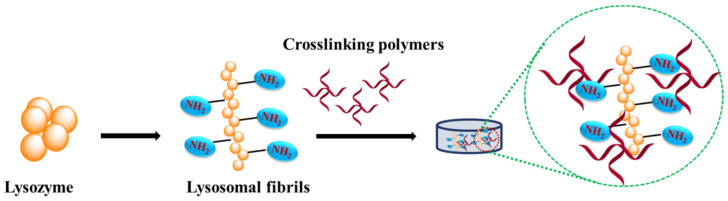
Approach towards lysozyme crosslinking for hydrogel formation.

Lysozyme is a naturally occurring protein belonging to the class of glycoside hydrolase and unanimously appears in diverse human tissues, including tears, saliva, and other body secretions [73]. Lysozyme plays a significant role in the innate protection system [74] and also exhibits antiviral, antiseptic, and anti-inflammatory features, making it a key aspirant in the pharmaceutical sector [75,76]. It is stable across various pH ranging from 5–9 and demonstrates a stable three-dimensional (3D) structure, make it a suitable candidate remodeling tissue microenvironment [77,78]. The approach in case of lysozyme crosslinking is presented in Figure 5. Lysozyme and its mutants have been used as crosslinking agents to synthesize different hydrogels for bone tissue engineering. In a study by Chen et al., a chitosan oligosaccharide-based hydrogel was prepared by crosslinking it with the help of T4 lysozyme mutant (T4M) [65]. The surface of T4M is rich in free amine groups, which function as efficient covalent crosslinkers, imparting good strength to the hydrogel network along with high specificity towards the binding of multivalent cations. This property was explored to exhibit localized delivery and the synergistic release of Mg^2+^ and Zn^2+^ for bone tissue repairment. Further, in order to improve the cellular affinity and rapid tissue regeneration, the integrin receptor-binding Arg-Gly-Asp (RGD) sequence was attached to the C-terminus of T4M. In another study, lysozyme-crosslinked 4-arm-PEG hydrogel was developed as a surgical sealant for tissue engineering [79]. The developed hydrogel could provide adequate mechanical stability responsible to endure high pressure. The in-situ formation of sealant to avoid fluid leakage is very important in medical sciences and can be utilized across many biomedical applications. The formation of hydrogels via lysozyme-based crosslinking is still under active exploration and has great potential. Rationally engineered lysozyme mutants have been reported to have enormous effect on hydrogel strength along with their enhanced cellular responses. The development of these variants could enhance the stability across a wide range of pH.

### 4.3. Horseradish Peroxidase

**Figure 6:**
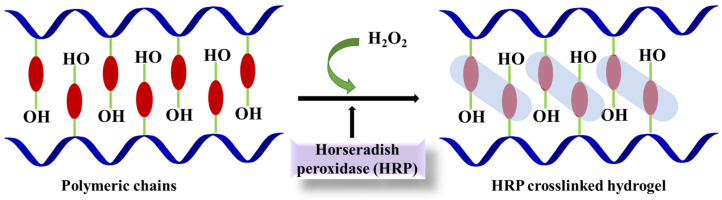
Mechanism of crosslinking via horseradish peroxidase for hydrogel formation.

Horseradish peroxidase (HRP) is a member of the peroxidase family and is known to be oxidoreductase with hydroperoxide as electron acceptor. HRP is a heme containing enzymes derived from horseradish roots and has a molecular weight of 40,000. HRP are commercially available in high purity form making it a representative system to investigate the structure, dynamic, and thermodynamic properties of peroxidases [80]. Several HRP isoenzymes have been reported with the most abundant and widely characterized C isoenzyme [81]. HRP crosslinked hydrogel have been widely reported as suitable for tissue engineering applications. HRP are capable of catalyzing the oxidative coupling of polymers-phenols in the company of hydrogen peroxide (H_2_O_2_), resulting in the formation of hydrogels with the tunable rate of gelation and crosslinking density [82] (Figure 6). The application of HRP has also been studied in tissue engineering; for the first time, Carnes et al. examined the HRP-catalyzed dityrosine-crosslinking of a fibrin scaffold for tissue engineering applications [83]. The work reported the effect of the concentration of HRP and H_2_O_2_ on the crosslinking density of fibrin microthreads, targeting its utility across a broad range of tissues for structural stability and mechanical strength. Further, Shoji et al. reported the in-situ formation of hyaluronic acid and tyramine-based hydrogel (HA-T) via taking the advantage of HRP in presence of H_2_O_2_ [84]. The fabricated hydrogel was loaded with bone morphogenetic protein (BMP)-2 and was examined for their ability to promote osteogenesis in a in vivo model. The study reported considerably greater bone volume, bone mineral content, and bone union at the fracture sites upon introduction with BMP-2-loaded HA-T hydrogel compared with the fractured sites with no treatment received.

**Figure 7:**
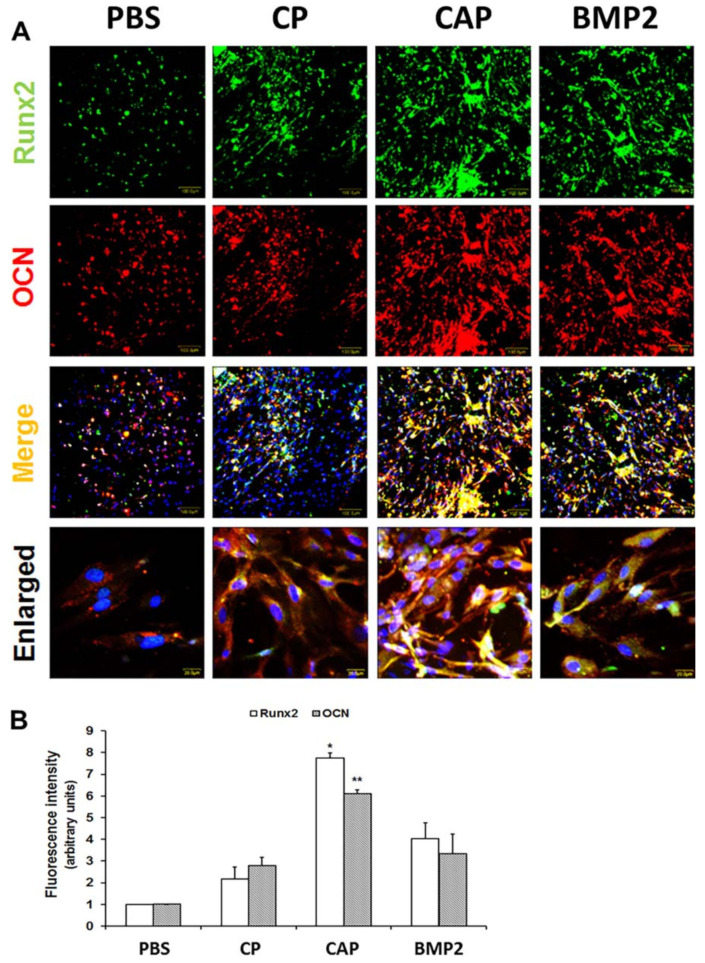
Expression of Runt-related transcription factor 2 (Runx2) and osteocalcin (OCN) in human PDLSCs (hPDLSCs) cultured in osteogenic medium (OM) for 14 days. (**A**): The hPDLSCs were cultured in hydrogels with calcium-accumulating peptide (CAP), control peptide (CP), or bone morphogenetic protein-2 (BMP-2) in four-well chambers and stained with respective antibodies. (**B**): (* *p*  <  0.05, ** *p*  <  0.05). Reproduced with permission from [85]. Copyright 2018 Wiley.

The loading and controlled release of bioactive agents, such as proteins and peptides, into hydrogels has been popular approaches in order to progress in the regenerative capacity of bone. In this regard, the research group of Park et al. synthesized bioactive calcium-accumulating peptide (CAP) comprising a collagen-binding design to accelerate osteogenic differentiation. Here, a gelatin-based hydroxyphenyl propionic acid hydrogel was fabricated with the help of an HRP-crosslinking approach in the presence of H_2_O_2_. Finally, CAP was chemically conjugated to the surface of the hydrogel. It was demonstrated that the presence of CAP-tagged hydrogel loaded with human periodontal ligament stem cells (PDLSCs) induced bone mineralization around the PDLSCs and also increased the expressions of the osteogenic markers at the in vitro level (Figure 7). The fabricated hydrogel system was able to recover a bone layer in a calvarial defect four weeks post-implantation [85]. Further, silk fibroin (SF)-based hydrogel has gained the considerable interest of researchers globally as a material of choice for tissue engineering applications. In this regard, Hasturk et al. fabricated SF which was enzymatically crosslinked with tyramine-substituted silk fibroin (SF-TA) or gelatin (G-TA)-hybrid hydrogels with the help of HRP and H_2_O_2_ [86]. Here, the manufactured hydrogel was chemically conjugated with RGD peptides. This approach gives precise control over the gelation of hydrogel along with adequate mechanical properties and bioactivity. In another study, the hydrogels of tyramine-modified gellan gum (Ty-GG) was manufactured by exploring both the physical and chemical method of crosslinking in the presence of HRP and H_2_O_2_ [87]. Here, the fabricated hydrogel was loaded with betamethasone, a potent drug to treat patients with rheumatoid arthritis. The sustained release of betamethasone in vitro, along with proliferation and inflammatory activity was also studied on chondrogenic primary cells and THP-1 cells. All these studies have presented a valuable approach for using HRP-based enzymatic crosslinking in the presence of H_2_O_2_ in order to synthesize in situ injectable hydrogels. A fast gelation rate along with control over the crosslinking density are some keynote features making HRP/H_2_O_2_ an attractive method for hydrogel crosslinking.

### 4.4. Transglutaminase (TG)

**Figure 8:**
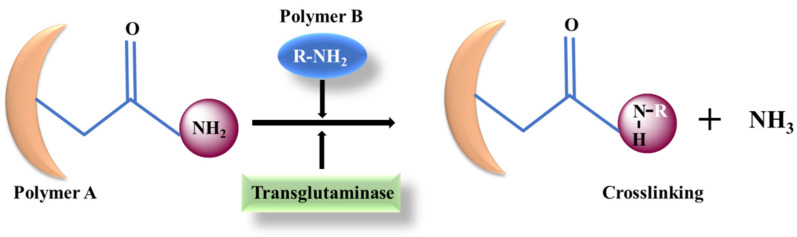
Mechanism of action of transglutaminase-mediated crosslinking strategy for hydrogel formation.

Transglutaminases, Ca^2+^-dependent acyl transferases, belong to the family of enzymes (EC 2.3.2.13) and are regarded as mild alternatives for chemical crosslinking strategies that catalyze the establishment of an amide bond between the γ-carbonyl and ε-amino groups of glutamine and lysine residues, respectively [88] (Figure 8). These enzymes have also been reported to contain an active site thiol group within a cysteine/histidine/aspartic acid (Cys-His-Asp) catalytic triad [89]. TG has been widely explored in enzyme-based crosslinkers for a wide range of polymers (such as gelatin, collagen, etc.) and proteins [90,91,92]. In fact, transglutaminase is emphasized as the best studied enzyme for protein-based hydrogel formation via enzymatic crosslinking for tissue engineering applications [21]. The adhesion supremacy and strong integration between the TG driven, in situ-formed hydrogel and the host tissue architect makes it a potent choice in for tissue regeneration process.

TG-crosslinked hydrogels have also been explored as implant coatings for the slow releasing of pharmaceutical agents. In this regard, Sun et al. fabricated TG-crosslinked gelatin-alginate hydrogel loaded with antibiotics (vancomycin) to prevent bacterial infection via the sustained release of vancomycin in tissue implants [93]. The study emphasized the release kinetics of loaded molecules as a function of the concentration of the enzymes used, as it is directly related to the degree of crosslinking. Further, the osteogenic potential of TG-crosslinked gelatin/hyaluronan in the presence of biotechnological chondroitin was explored by La Gatta et al. [94]. In this study, a semi-interpenetrating gel was formed with high stability, improved stiffness, and lower swelling extent compared to native gelatin hydrogel. The effect of formed hydrogel on bone regeneration was evaluated via the assessment of the osteogenic differentiation by seeding the hydrogel with human dental pulp stem cells. Over a period of 30 days, the upregulation of the expression of both osteocalcin and osteopontin at gene and protein level was observed.

The understanding of the interplay between the bone marrow microenvironment and resident cells is very important and has been explored as a vital tool with substantial clinical value [95,96,97]. Fundamentally, the biophysical and biochemical aspects across the bone marrow niche play a profound role in the complete functioning of the organ [98]. In this regard, Vallmajo-Martin et al. fabricated a TG-crosslinked hybrid hydrogel system comprising poly (ethylene glycol) (PEG) and hyaluronic acid (HA) for the formation of bone marrow analogues [99]. In this work, it was demonstrated that the fabricated hybrid hydrogel was able to maintain, inflate, and differentiate human bone marrow-derived stromal cells and human hematopoietic stem and progenitor cells in vitro. This hydrogel could serve as an ideal scaffold for various tissue engineering applications. For an ideal hydrogel, the concentration of the initial molecules and the enzyme is very crucial, and its ratio will decide the degree of crosslinking and the crosslinking density, as both factors are very critical in the formation of scaffolds for tissue engineering applications.

### 4.5. Alkaline Phosphatase (ALP)

**Figure 9:**
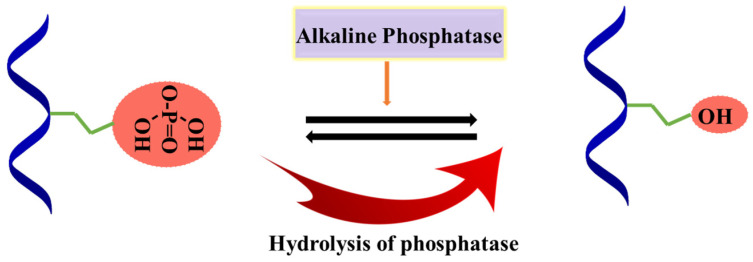
Cleavage of phosphate group from organic phosphate in presence of ALP.

ALP plays a crucial role in the mineralization of bone and works by cleaving phosphate from organic phosphate [100]. Figure 9 represents its mechanism of action, where the hydrolysis of phosphate occurs upon the action of ALP in the presence of organic phosphate group.

**Figure 10:**
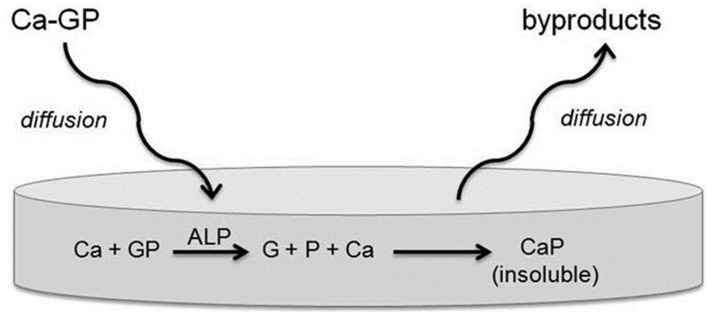
Basic principle of mineralization by entrapped ALP. Calcium glycerophosphate (Ca-GP) diffuses into hydrogels with incorporated ALP. Inside the hydrogel, glycerophosphate (GP) is cleaved to phosphate (P) by ALP, which reacts with Ca^2+^ ions to form insoluble CaP. Reproduced with permission from [102]. Copyright 2012 Wiley.

Owing to their participation in the mineralization of skeletal tissues, ALP has been explored as an outstanding supplier of the phosphatase for hydrogel formation [101]. In the field of bone tissue engineering, the utility of ALP-crosslinked hydrogels is to encourage homogeneity in the mineralization process of hydrogels, which in turn is crucial for imparting the mechanical strength or bestow them more suitability in the area of bone replacements. In this direction, Douglas et al. fabricated three ALP-crosslinked hydrogel systems, namely, mussel protein-inspired catechol-PEG (cPEG), type I collagen, and oligo PEG fumarate (OPF), for bone tissue replacements and to induce mineralization via calcium phosphate (CaP) [102]. The aim of this study was to inspect the retention power of ALP in these hydrogels along with the initiation of its mineralization (Figure 10). Additionally, the nature and amount of the mineral formed in the hydrogel systems along with the effect of mineralization on the morphology and mechanical stability of the synthesized hydrogels were also investigated.

Up recently, supramolecular hydrogels have gained substantial attraction in the field of tissue engineering, cancer therapy, and drug delivery [103]. In this line, Yuan et al. fabricated an ALP-crosslinked hydrogel series using the self-assembly capability of the core segment (GNNQQNY) of the yeast prion Sup35 [104]. The study highlighted the ability of ALP to promote the functioning of precursors, such as hydrogelator, which then self-assembles in an aqueous environment to form nanofibers (width ≤ 10 nm). This work could be implemented in bone tissue engineering to promote self-assemblies-based hybrid hydrogels. In order to fabricate and efficient hybrid hydrogel system using ALP-crosslinking, the ratio of the formed minerals and the utilized polymer networks is extremely critical, along with the ALP concentration-dependent mineralization rate. Optimal conditions, together with the thorough regulation of governing parameters, could provide a hybrid hydrogel to induce and accelerate bone mineralization, which serves as an alternative to including calcium phosphate in the designed system.

The development and clinical translational aspect of bone tissue engineering requires the careful administration of encapsulated biomolecules/pharmaceutical excipients inside the hydrogel scaffolds. In order to achieve this, regulation in the pore size, pore arrangement and pore capacity of the designed hydrogel plays a substantial role. The design of hydrogel plays a vital role in regulating the cell attachment and its proliferation. Researchers have also emphasized the accountability of the functionally graded scaffold (FGS) and non-functionally graded scaffold (NFGS) as functions of computational dynamics for permeability analysis [105,106]. A crosstalk between different fields, including but not limited to computational science, materials sciences, nanotechnology, tissue engineering, etc., provides an innovative and comprehensive approach to envisage our vision and understanding in the vast field of science. A table summarizing the different studies on enzyme-based crosslinking in hydrogel formation-targeting bone tissue engineering is presented in Table 2.

## 5. Conclusions and Future Prospects

To pacify the growing exigency of biocompatible and biodegradable scaffolds for bone tissue repair and clinical translations, hydrogels are an exceptional topic. In this context, enzyme-based crosslinking approaches for hydrogel fabrication is a relatively new and upcoming concept. As pointed, the specificity and high catalytic efficiency of enzymes have provided a podium for the in-situ gelation of macromolecules with high efficiency. The minimal enzyme requirement as a crosslinking agent, along with negligible toxicity of the formed by products, are other benefits that marks the supremacy of this technique. Enzymes are biological molecules, which make them biocompatible, a key requirement in the clinical translation of fabricated materials/systems for biomedical applications. However, the high processing cost of enzymes still remains an obstacle that hinders the progression and global recognition of this approach. Currently, there are still lot of blind spots in the field of enzyme-mediated hydrogel fabrication that need serious considerations, such as the recovery of the enzymes after the completion of the process. The storage of hydrogels is another tricky aspect that impedes their clinical application. To tackle these concerns, engineered enzymes are being developed with minimal requirements and high catalytic efficiency. Additionally, the use of nanoparticles for enzyme recovery during the process is another aspect that has acquired substantial attention. The field of enzyme-mediated hydrogel synthesis is rapidly maturing and, in the near future, innovative approaches using enzymes-based hydrogel manufacturing will surface and continue to flourish for clinically oriented hybrid hydrogel designing for bone tissue engineering.

## Figures and Tables

**Table 1:** Potential physical and chemical crosslinking approaches for creating hybrid hydrogels.

Type of Crosslinking	Mechanism/Response	Advantages	Disadvantages	Ref.
Physical approaches				
Hydrogen bonding	Interaction between electropositive (H) and electronegative (O, N, F) atoms	Safe and less toxic, non-covalent bonds (sacrificial), highly dynamic and reversible character, excellent toughening effect, lowest toxicity, tunable properties, and allows deviations in ion amounts	Weak mechanical characteristics, highly detrimental in aqueous medium, easily dissociate upon heating, low degree of crosslinking	[33,34,35]
Electrostatic (ionic) interactions	Interaction between positively charged ions (Ca^2+^, Mg^2+^, Cu^2+^, etc.) and negatively charged functional groups or interaction between anionic and cationic molecules or polyelectrolytes under physiological environment	Safe and less toxic, In-situ formation, highly dynamic and reversible character, excellent tunability, stimuli-sensitive behavior	Weak mechanical characteristics, low degree of crosslinking	[36,37]
Hydrophobic/hydrophilic interactions	Interaction between chemical groups located close to one another	Safe and less toxic, sol-gel transition (i.e., thermal gelation), highly dynamic and reversible character, and tunable properties	Low mechanical properties, hydrophobic/hydrophilic balance is sensitive to polar solvents and temperature, incapability to control the reaction growth, low degree of crosslinking,	[38,39]
Entanglements of polymeric chains	Complication of intramolecular or intermolecular chains.	Safe and less toxic, enhanced interchain connectivity	Too weak, depends on molecular weight fraction of polymers	[40,41]
Stereocomplex crystallization	Highly organized form of atoms or molecules/parallel or antiparallel interactions between two independent helices or co-crystallization between polymers with complementary configurations	Safe and less toxic, alternative packing of different polymer chains, and tunable and better mechanical properties		[42,43]
Metal coordination	A coordination complex of a central atom/ions (typically metallic) and surrounding arrangement of bound ligands	Safe and less toxic, complexation between ligand-adorned polymers and metal ions	Low degree of crosslinking	[44,45,46]
Stacking of π–π bonds	Speculative attractive and noncovalent (orbital overlapping) interface between the π bonds in aromatic rings	Strong physical interaction (noncovalent), non-destructive and reversible, can provide hydrophobic domain	Low degree of crosslinking, not very weak interactions, not very directional, generally aromaticity is essential π–π stacking interactions, sensitive to polar solvents, non-negligible cytotoxicity	[47,48,49]
Chemical approaches				
Enzymatic effect	Enzyme catalyzes the functional groups of the substrate in close proximity	Highly biocompatible, high specificity (chemo-, region-, and stereo-selectivity), high crosslinking density, and minimal requirement	High processing cost, negligible recovery, high sterile condition required, high degree of crosslinking,	[24,50]
Schiff-base reaction (imine formation)	Shortening of primary amines and active carbonyl groups (aldehyde or ketone) to form imine bonds	Dynamic reversibility, potential pH-sensitive linker, in situ formation of hydrogels	Required washing to remove remaining unreacted substances, high degree of crosslinking	[51,52]
Michael type addition reaction	Reaction between nucleophile and crosslinked unsaturated carbonyl systems (e.g., olefins, alkynes) to form (-C–C-) bond	In situ formation of hydrogels	Required washing to remove remaining unreacted substances, high degree of crosslinking	[53,54]
Oxime bond formation	Reaction between an aldehyde or ketone and hydroxylamine	Dynamic covalent character, high efficiency, chemo-selectivity, formation in aqueous solvents and water being the only side-product	Complex synthetic methods, Required washing to remove remaining unreacted substances, high degree of crosslinking	[55,56]
Diels–Alder “click” reaction	No catalyst needed	Dynamic covalent character, mild and copper-free reaction, facile for in vitro and in vivo applications,	Long gelation time relatively, Required washing to remove remaining unreacted substances, high degree of crosslinking	[57,58]
Free-radical polymerization	Production of free radicals in monomers using particular initiator and then they form long polymeric chains by joining them together through covalent bonding	Rapid and simple method, no sophisticated and expensive instruments needed	Difficult to control chain propagation, wide particle size distribution, required washing to remove remaining unreacted substances, high degree of crosslinking	[59,60]

**Table 2:** Summary of different studies on enzyme-mediated crosslinking hydrogels for bone tissue engineering.

S. No	Enzyme Involved/ Mimicked	Composition	Key Features	Ref.
1.	Tyrosinase	Hyaluronic acid, Chitosan, Dopamine hydrochloride	Multilayer membrane formation, Enhanced cell adhesion, viability, proliferation, and density of immortalized murine calvarial cell line (MC3T3-E1)	[66]
2.	Tyrosinase	Carboxymethyl–chitosan (CMC), Gelatin, Nano-hydroxyapatite	Assessment of CMC-dependent crosslinking and strength; support in proliferation and differentiation of osteoblast; gel stability in vivo	[67]
3.	Tyrosinase	Silk fibroin, Gelatin, Calcium chloride	Enhanced the osteogenesis of bone marrow derived progenitor cells (hMSCs); upregulating the gene expression of osteogenic markers (RUNX2, COL I, ALP, OPN, ON); osteocytic markers (DMP 1, PDPN, SOST), and β-catenin, BMP-2, and BMP4; enhancing mineralization processes	[68]
4.	Tyrosinase	Chitosan, Gelatin, Nanohydroxyapatite	Pore size greater than 150 µm; high swelling ratio; acceptable biodegradability and biocompatibility	[71]
5.	Tyrosinase	Polycaprolactane, beta-tricalcium phosphate	Enhanced viability and proliferation of osteoblast cells; development of an osteo-mucosal model; stable adhesive strength in wet conditions	[72]
6.	Lysozyme	Polyethylene Glycol (PEG)	Facile clinical process; good biocompatibility; tight adherence to tissues	[79]
7.	Horseradish peroxidase (HRP)	Hyaluronic acid, Tyramine	In situ-formed hydrogels; sustained release of BMP-2 in vitro; enhanced gene expressions of osteogenic makers, Alpl, Bglap, and Osx; callus formation in fractured bone in vivo	[84]
8.	Horseradish peroxidase (HRP)	Calcium-accumulating peptide, Collagen	Induced bone mineralization around the human periodontal ligament stem cells (PDLSCs) loaded in hydrogel; increased osteogenic marker expressions in vitro; formation of bone layer post implantation in vivo	[85]
9.	Horseradish peroxidase (HRP)	Silk fibroin, Gelatin	Enhanced gelation kinetics; improved mechanical attributes; slow enzymatic degradation; imparted stiffness and β sheet formation; superior morphology and metabolic activity of human mesenchymal stem cells (hMSCs)	[86]
10.	Horseradish peroxidase (HRP)	Tyramine, Gellan gum	High degree of substitution (~30%); sustained release of betamethasone; high mechanical strength; negligible cytotoxicity and non-significant change in the metabolic activity of chondrogenic primary cells	[87]
11.	Transglutaminase	Gelatin, Alginate	Cost-effectiveness and adequate safety profile; offered antibacterial properties; effective in reducing implant-related infection; upon treatment in vivo, substantial higher bone volume with intact bony architect was observed	[93]
12.	Transglutaminase	Gelatin, Hyaluronan, Bacteriological chondroitin,	Enhanced expression of osteocalcin, and osteopontin at gene and protein level in human dental pluripotent stem cells (hDPSCs); effective in accelerated bone regeneration	[94]
13.	Alkaline phosphatase	Calcium phosphate, Collagen type-1, catechol substituted PEG (cPEG), Fumaric acid/PEG copolymer (OPF)	The effectiveness in mineral formation decreases in the order cPEG > collagen > OPF; highest increment of Young’s modulus in cPEG was demonstrated	[102]
14.	Alkaline phosphatase	Yeast prion Sup35 with the insertion of peptide segment (GNNQQNY)	Self-assembled nanofibers formation of hydrogelators; cytocompatible upon the insertion of the peptide segment along with enhancement in self-assembly	[104]

## Data Availability

Not applicable.
